# Hypoxia-induced TET1 facilitates trophoblast cell migration and invasion through HIF1α signaling pathway

**DOI:** 10.1038/s41598-017-07560-7

**Published:** 2017-08-14

**Authors:** Jingping Zhu, Kai Wang, Ting Li, Jiayu Chen, Dandan Xie, Xinwen Chang, Julei Yao, Jinting Wu, Qian Zhou, Yuanhui Jia, Tao Duan

**Affiliations:** 1 Clinical and Translational Research Center, Shanghai First Maternity and Infant Hospital, Tongji University School of Medicine, Shanghai, 200040 China; 2 Department of Obstetrics, Shanghai First Maternity and Infant Hospital, Tongji University School of Medicine, Shanghai, 200040 China

## Abstract

Low oxygen is a typical extrinsic factor for the regulation of trophoblast biological function, including cell migration, invasion and proliferation. Ten-eleven translocation methylcytosine dioxygenase 1 (TET1), an enzyme converting 5-methylcytosine (5-mC) to 5-hydroxymethylcytosine (5-hmC), is transcriptionally activated by hypoxia in cancer cells. Therefore, we focus on the role of TET1 on trophoblast function in a physiologically hypoxic environment (3% oxygen), which is related to early placentation. Here, we found that TET1 was highly expressed in first trimester villi compared with normal term placentas. *In vitro*, both TET1 mRNA and protein expression levels in JEG3 cells were increased following exposure to 3% oxygen, and the migration and invasion capacities of JEG3 cells were up-regulated. Furthermore, TET1 knockdown decreased the migration, invasion and proliferation of JEG3 cells exposed to 3% oxygen, and the expression of HIF1α and its downstream target genes was also decreased, which was related to hyper-methylation of the HIF1α promoter. Finally, increased HIF1α protein expression reversed the inhibitory effect of TET1 knockdown on the migration and invasion of JEG3 cells exposed to 3% oxygen. These data show that hypoxia-induced TET1 expression facilitates trophoblast cell migration and invasion through the HIF1α signaling pathway, which plays an important role during placentation.

## Introduction

In early pregnancy, extra villous cytotrophoblast cells occlude the uterine spiral artery. During this period, there is little maternal blood in the inter-villous space (IVS). However, at approximately 10–12 weeks of gestation, the cytotrophoblasts replace the maternal endothelial cells and the spiral arterioles are remodeled, which creates large-diameter, low-resistance vessels carrying more blood to the maternal-fetal interface^1^. These physiological changes among virtually all of the 100 to 150 spiral arteries in the placental bed are necessary for a successful pregnancy^2^. Therefore, initially the placenta develops in a low oxygen (O_2_) environment of 2–3% O_2_ until the 10th week of pregnancy^3^. Because of the low level of anti-oxidant enzymes in the placenta, changes in O_2_ tension within the IVS play a physiological role in trophoblast differentiation and invasion, which must be strictly controlled due to their relation to pregnancy outcomes^4^. Although earlier *in vitro* studies by Genbacev *et al*. reported that hypoxia inhibited the invasion of primary trophoblast cells^5, 6^, in recent years, studies in trophoblast-like cell lines such as HTR-8/SVNEO and JEG3 cells showed that genes related to cell migration and invasion were highly expressed^7^, and *in vitro* cell migration and invasion capacities were up-regulated under low O_2_ environments^8–10^.

Hypoxia is a pervasive stimulus that affects a wide variety of biological processes. Hypoxia induces the nuclear translocation and dimerization of hypoxia-inducible factor alpha (HIFα) with HIFβ (ARNT), forming HIF (HIF1, HIF2 and HIF3), followed by binding to the hypoxic response element of related genes^11^. The target genes are involved in glycolysis, red blood cell production and angiogenesis. The HIF1α and HIF2α proteins are constitutively expressed in the human placenta^11^, with peak mRNA and protein levels of HIF1α observed at 7–10 weeks of gestation^12^; however, few studies have examined HIF3 expression in the placenta. Studies have shown that placentas from Arnt^−/−^, Hif1α^−/−^ or Hif2α^−/−^ embryos exhibit defective trophoblast invasion and placental vascularization, resulting in aberrant cell fate adoption^13^.

The ten-eleven translocation (TET) proteins convert 5-methylcytosine (5-mC) to 5-hydroxymethylcytosine (5-hmC), which is an important DNA demethylation mechanism^14, 15^. TET proteins can also catalyze 5-mC to 5-formylcytosine and 5-carboxycytosine^16^. Three members of the TET family have been identified: TET1, TET2, and TET3. Koh *et al*. found that TET is highly expressed in embryonic stem (ES) cells^17^, and TET expression is up-regulated during the generation of induced pluripotent stem cells^18^. Genome-wide studies in undifferentiated ES cells and during ES cell differentiation have provided detailed information regarding the distribution of Tet1 and 5 hmC, and Tet2 plays an important role during the pluripotent state and initiation of cellular differentiation. The successive expression of Tet1 and Tet2 play complementary roles during cell differentiation. In addition, decreased expression of TET1 and TET2 and changes in the level of 5-hmC are thought to be associated with the onset and progression of several types of cancer. These studies demonstrate that the TET family plays important roles in ES cell differentiation, development, and transformation and that TET1 and TET2 play similar roles.

The major epigenetic mechanisms include DNA methylation, histone modifications, and genomic imprinting. To adapt to hypoxia, global hypo-methylation plays a central role in hypoxic response pathways in tumor cells^19–21^. Moreover, recent studies have demonstrated that TET1 is up-regulated under hypoxic conditions and plays a role in regulating the transcriptional response to hypoxia in cancer^22, 23^. However, the function of TET1 during placentation has been rarely studied.

In this study, we sought to elucidate the biological function and mechanism of TET1 in trophoblast cells exposed to 3% O_2_. We compared TET1 expression in villi and term placentas. Using a transwell migration/invasion assay, we investigated the role of TET1 in trophoblast cell migration and invasion following exposure to 3% O_2_. The results showed that the expression of TET1 is higher in villi than in term placentas. The qPCR and western blotting analyses revealed that TET1 knockdown inhibits cell migration and invasion, which was followed by a decrease in HIF1α expression due to hyper-methylation of the HIF1α promoter. In addition, the TET1 knockdown-induced inhibition of cell migration and invasion in trophoblast cells exposed to 3% O_2_ was reversed by increasing HIF1α expression.

## Results

### TET1 is highly expressed in first trimester villi

**Figure-1:**
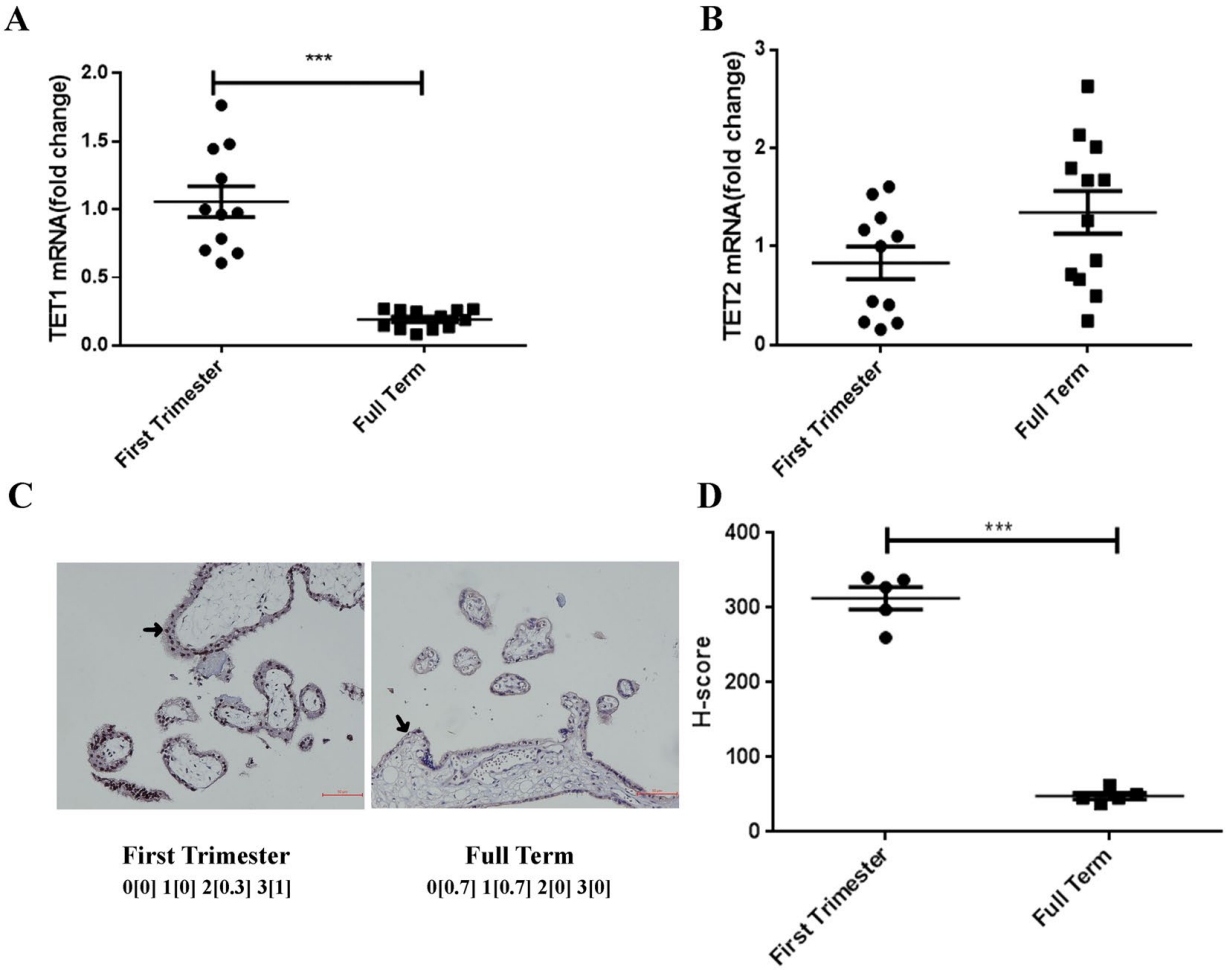
TET1 is highly expressed in first trimester villi. The mRNA expression of TET1 (**A**) and TET2 (**B**) in first trimester villi (7.43 ± 0.29 weeks, n = 11) and normal full term placentas (39.06 ± 0.11 weeks, n = 12) was detected by qPCR. (***P < 0.001, Student’s t-test) (**C**) The first trimester villi showed a high percentage of 2 + (15% equals 0.3) and 3 + (85% equals 1) staining, whereas the full term placentas had no (60% equals 0.7) and 1 + (40% equals 0.7) TET1 staining, as analyzed using IHC. (Asterisk, trophoblast cell) (**D**) H-score for the TET1 staining between the first trimester villi and normal full term placentas. (n = 5, ***P < 0.001, Student’s t-test).

The expression of TET1 and TET2 was investigated by qPCR analysis and we found that TET1 (Fig. 1A), but not TET2 (Fig. 1B), was significantly elevated in first trimester villi compared with normal full term placentas. We also performed immunohistochemistry (IHC) to localize TET1 protein expression in the human placenta, which demonstrated that TET1 was mainly expressed in the nuclear of trophoblasts and that trophoblast TET1 expression was much higher in first trimester villi than in full term placentas (Fig. 1C,D).

### TET1 expression is increased in JEG3 cells exposed to 3% O_2_

**Figure-2:**
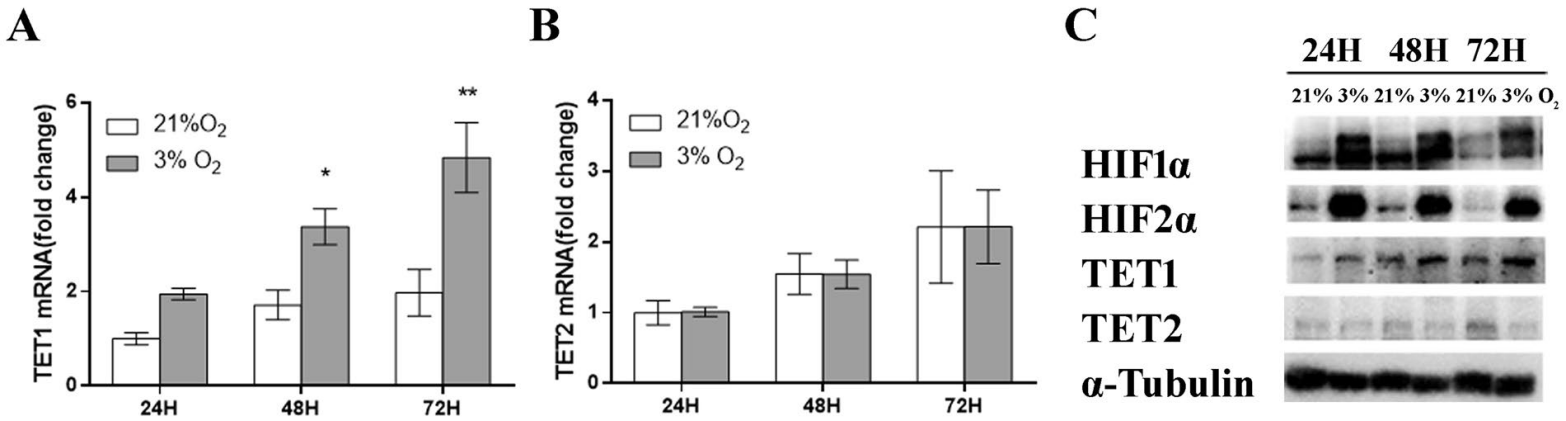
TET1 mRNA and protein expression is increased in cells exposed to 3% O_2_. JEG3 cells were cultured in 3% or 21% O_2_ for 24 h, 48 h and 72 h. TET1 (**A**) and TET2 (**B**) mRNA expression was analyzed using qPCR. (*P < 0.05, **P < 0.01, one-way ANOVA) (**C**) The protein expression of HIF1α, HIF2α, TET1 and TET2 was detected by western blotting. α-Tubulin was used as the internal control.

In early pregnancy, physiologically low levels of O_2_ regulate the expression of multiple genes responsible for the maintenance of normal pregnancy. Both TET hydroxylase and DNA demethylation have essential roles in the regulation of gene expression. To determine whether TET levels are altered in 3% O_2_, JEG3 cells were incubated in 3% O_2_ or 21% O_2_. The observed results were consistent with previous findings in placental tissues. The expression of TET1 (Fig. 2A), but not TET2 (Fig. 2B), was elevated in cells exposed to 3% O_2,_ which was also confirmed using western blotting (Fig. 2C). Furthermore, the expression of the TET2 protein decreased as the expression of TET1 increased, suggesting that the effects of TET1 and TET2 are complementary, as previously reported. As a control for hypoxia, we detected the protein expression of HIF1α and HIF2α. These findings indicate that both the mRNA and protein expression levels of TET1 are increased in JEG3 cells exposed to 3% O_2_.

### JEG3 cell migration and invasion are up-regulated in cells exposed to 3% O_2_, whereas proliferation is inhibited

**Figure-3:**
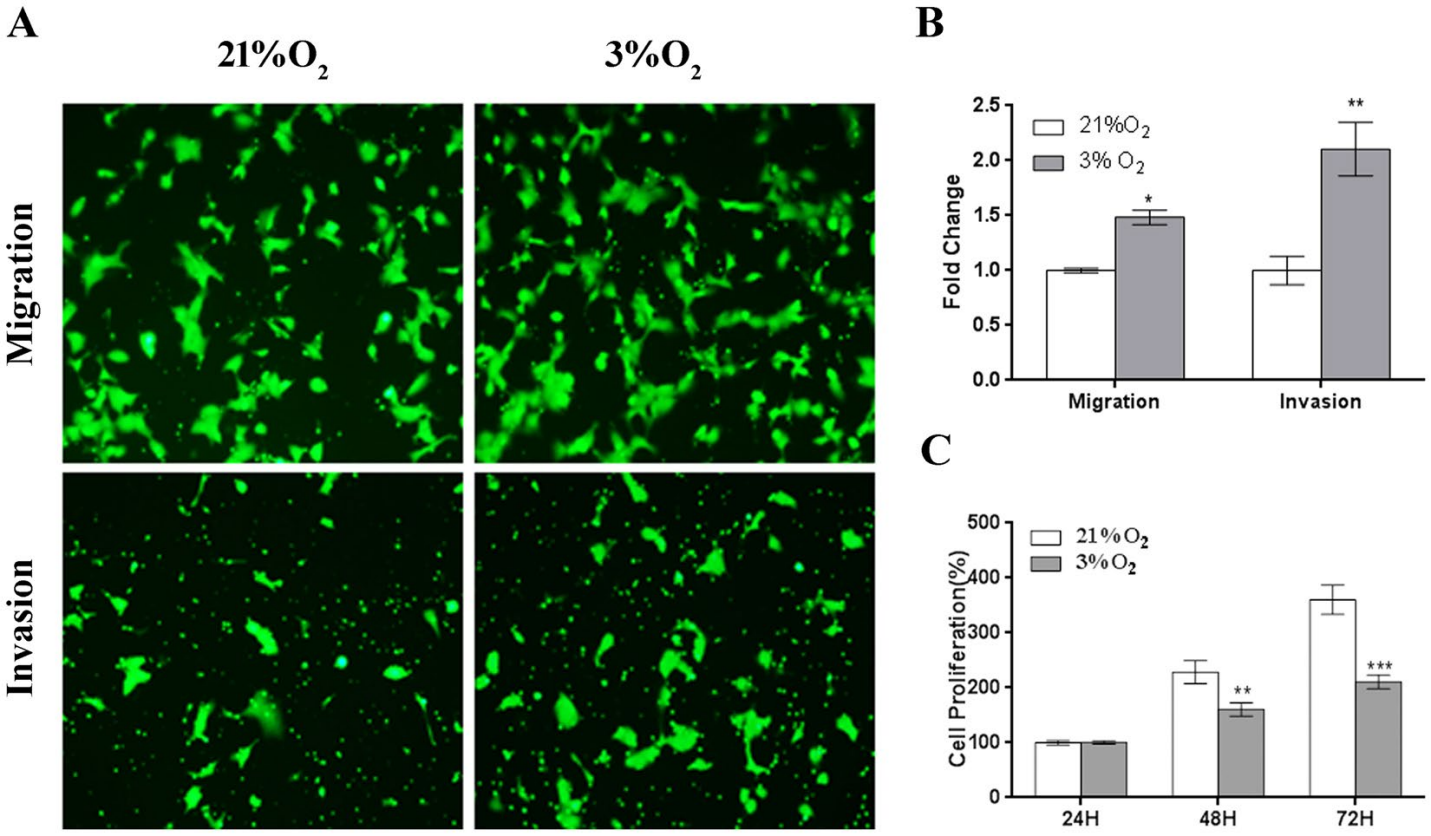
The migration and invasion capacities of JEG3 cells are up-regulated following exposure to 3% O_2_, whereas the proliferation capacity is inhibited. (**A**) Images depicting the migration and invasion of JEG3 cells exposed to 3% or 21% O_2_. (**B**) The fold change in the cell number relative to the control (21% O_2_) from three independent experiments. The quantitative data are expressed as the mean ± S.E.M. (*P < 0.05, **P < 0.01, Student’s t-test) (**C**) The proliferation capacity of JEG3 cells exposed to 3% or 21% O_2_ for 72 h. (**P < 0.01, ***P < 0.001, one-way ANOVA).

The O_2_ concentration has major effects on trophoblast biological functions, including cell migration and invasion. Therefore, we detected the migration and invasion of JEG3 cells exposed to 3% O_2_ using the transwell migration/invasion system_._ As a control, the migration and invasion assays were also performed in JEG3 cells exposed to 21% O_2_. We found that cell migration and invasion were increased following exposure to 3% O_2_ (Fig. 3A,B), indicating that cell migration and invasion were up-regulated under hypoxic conditions_._ In contrast_,_ cell proliferation was decreased in cells exposed to 3% O_2_ (Fig. 3C). These results demonstrate that the migration and invasion capacities of JEG3 cells are up-regulated following exposure to 3% O_2,_ and that this effect is not dependent on increased cell proliferation.

### The migration and invasion capacities of JEG3 cells are more significantly decreased by TET1 knockdown in cells exposed to 3% O_2_ than in cells exposed to 21% O_2_

**Figure-4:**
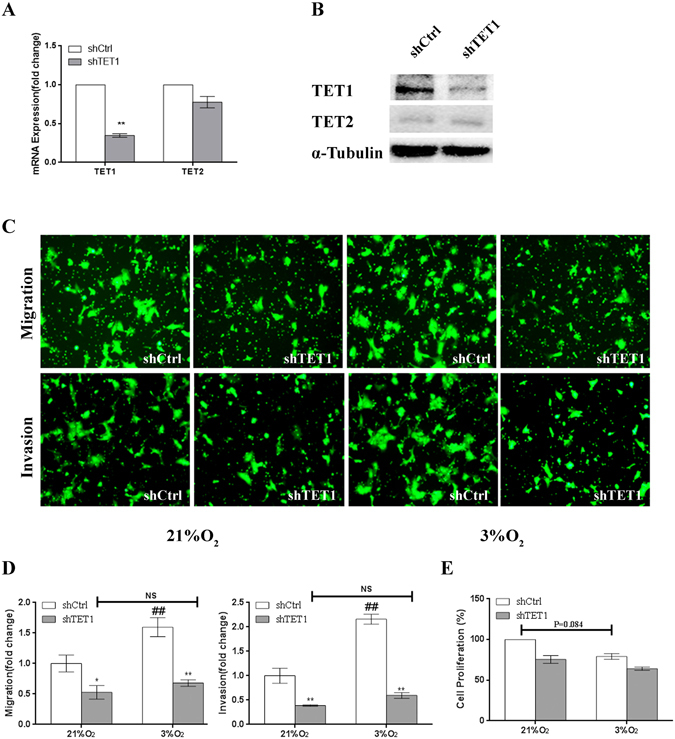
The migration and invasion capacities of JEG3 cells are more significantly suppressed by TET1 knockdown in cells exposed to 3% O_2_ than in cells exposed to 21% O_2_. (**A**,**B**) Real-time q PCR and western blot analyses showing the specific knockdown of TET1 in JEG3 cells by shRNA. (**C**) The migration and invasion capacities of JEG3 cells transfected with scramble shRNA or TET1 shRNA and then exposed to 21% or 3% O_2_ were assessed using the transwell assay. (**D**) The migrated or invaded cells were counted and the quantitative data are expressed as the fold change from the control of three independent experiments. (*P < 0.05, **P < 0.01, shCtrl vs. shTET1; ^##^P < 0.01 compared to shCtrl in 21% O_2_; there was no significant difference between the shTET1 cells exposed to 3% and 21% O_2_, one-way ANOVA) (**E**) Proliferation capacity of JEG3 cells transfected with scramble shRNA or TET1 shRNA and then exposed 3% O_2_. (P = 0.0.84, between the shCtrl cells exposed to 3% and 21% O_2_, one-way ANOVA).

We showed that 3% O_2_ promotes JEG3 cell migration and invasion and that TET1 is highly expressed in cells exposed to 3% O_2_. Therefore, we hypothesized that TET1 plays a role in regulating cell migration and invasion following exposure to 3% O_2_, the physiological O_2_ level in early placentation. Hence, we generated JEG3 cells that stably expressed a TET-targeting short hairpin RNA (shRNA; shTET1). Using qPCR (Fig. 4A) and western blotting (Fig. 4B), we confirmed the efficiency of TET1 knockdown and confirmed that it did not interfere with TET2 accumulation. Then, we detected cell migration, invasion and proliferation in JEG3 cells expressed shCtrl or shTET1_._ The cell migration and invasion were down-regulated by TET1 depletion in the cells exposed to either 21% or 3% O_2_, which was more significant in the cells exposed to 3% O_2_ (Fig. 4C,D) and there was no difference between the 21% and 3% O_2_ group in the TET1 knockdown cells. Therefore, we hypothesized that TET1 is necessary for the hypoxia-induced up-regulation of cell migration and invasion. Similarly, we found that the TET1 knockdown cells had lower proliferation rates exposed to either 21% or 3% O_2_ (Fig. 4E).

### TET1 regulates the expression of HIF1α and the hyper- or hypo-methylation status of the HIF1α promoter region in cells exposed to 3% O_2_

**Figure-5:**
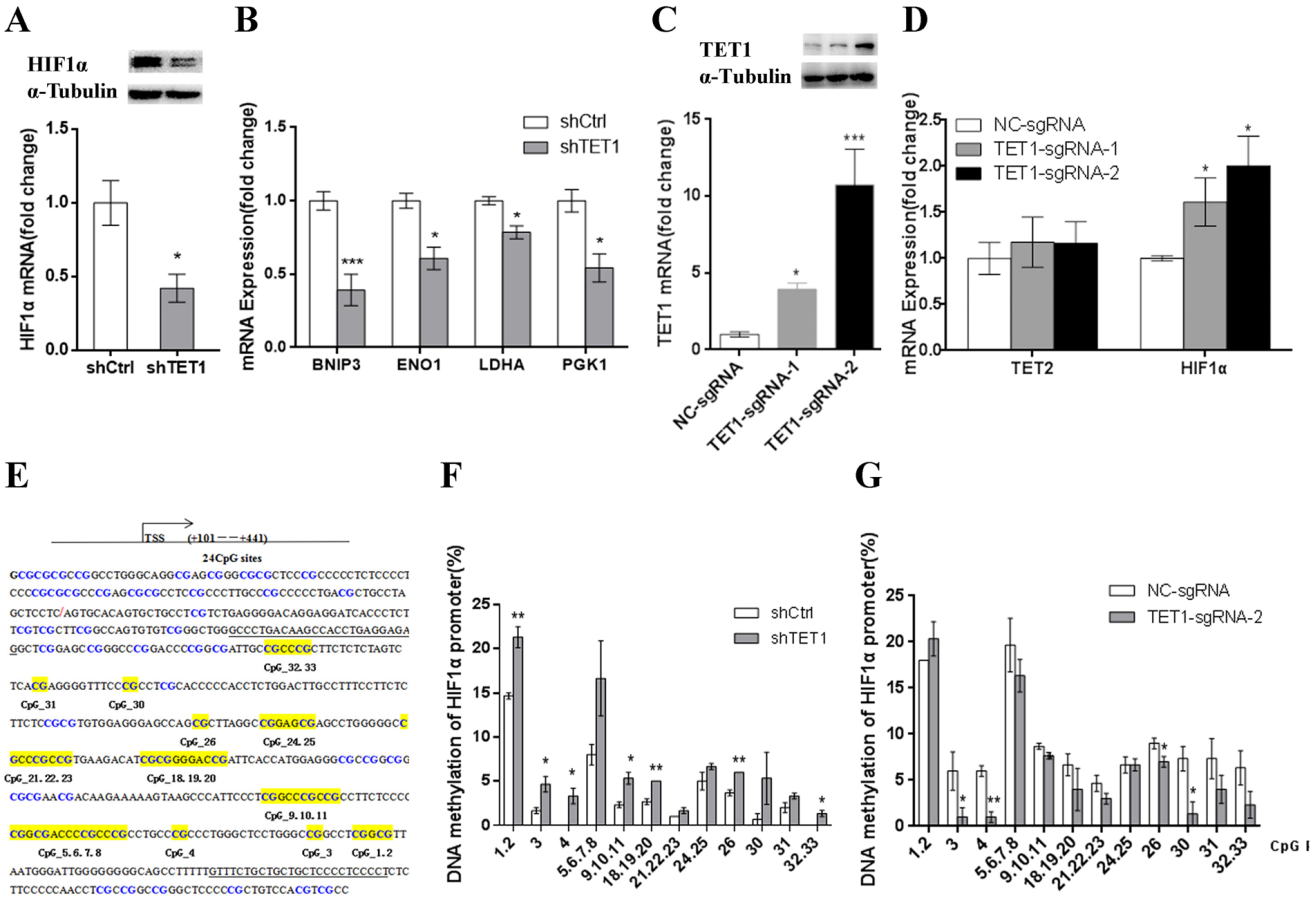
TET1 regulates the expression of HIF1α following exposure to 3% O_2_, which is accompanied by changes in the hyper- or hypo-methylation status of the HIF1α promoter. (**A**) The mRNA and protein expression of HIF1α in TET1 knockdown JEG3 cells exposed to 3% O_2_ for 48 h. (**B**) After TET1 knockdown in JEG3 cells exposed to 3% O_2_ for 48 h, the mRNA expression of the HIF1Aα target genes BNIP3, ENO1, LDHA and PGK1 was altered. (**C**) qPCR and western blotting analyses showing the overexpression efficiency of TET1 in JEG3 cells transected with sgRNAs. (**D**) The mRNA expression of TET2 and HIF1α in TET1-overexpressing JEG3 cells exposed to 3% O_2_ for 48 h. (**E**) The HIF1α promoter region. The blue color indicates the CpG sites. The 24 CpG sites shaded in yellow were divided into 12 units for the analysis. TSS is labeled by the red oblique line. The primer region is underlined. (**F**,**G**) DNA methylation of the HIF1α promoter at different CpG units was detected in JEG3 cells cultured in 3% O_2_ for 48 h using MALDI TOF mass spectrometry. (*P < 0.05, **P < 0.01, Student’s t-test).

Previous studies have shown that TET1 is required for the induction of hypoxia-responsive genes in neuroblastoma^22^ and may act as the co-activator of HIF1α, which participates in hypoxia-induced migration and invasion^23^. HIF1α plays an important role under hypoxic conditions; therefore, we examined the mRNA and protein expression of HIF1α in TET1-knockdown cells exposed to 3% O_2_. We found HIF1α mRNA expression was significantly down-regulated by the depletion of TET1, which was also confirmed by western blotting (Fig. 5A). In cells exposed to 3% O_2_ for 48 h, the expression of the HIF1α target genes BNIP3, PGK1^22^ and LDHA^23^, which have been previously shown to be regulated by TET1, was altered. In addition, the expression of ENO1, which functions as a glycolytic enzyme, was also altered in the TET1 knockdown cells exposed to 3% O_2_ (Fig. 5B). To further test whether TET1 impacts the expression of HIF1α, we generated a TET1-overexpressing cell line using single guide RNAs (sgRNAs) to target nuclease-dead Cas9-mediated transcriptional activation^24^. TET1 overexpression (TET1-sgRNA-1 and TET1-sgRNA-2) in JEG3 cells resulted in significantly increased TET1 mRNA expression compared with the negative control group (NC-sgRNA), which was also confirmed using western blotting (Fig. 5C). In addition, the overexpression of TET1 up-regulated the HIF1α mRNA level in cells exposed to 3% O_2_ (Fig. 5D). But the mRNA expression of HIF1α was not regulated by TET1 in cells exposed to 21% O_2_ (data shown in Fig. S1).

Several studies have demonstrated that TET1 can bind to the rich CpG regions to regulate gene expression^25, 26^ and that TET1 depletion results in hyper-methylation of the DNA of target genes through a significant decrease in 5-hmC. Therefore, we hypothesized that decreased expression of HIF1α induced by the loss of TET1 was the result of DNA hyper-methylation. To test this hypothesis, we performed a pilot study to evaluate the differences in DNA methylation of the HIF1α promoter between the shCtrl and shTET1-transduced JEG3 cells exposed to 3% O_2_. As shown in Fig. 5E, the CpG sites of the HIF1α promoter were enriched. We found that most of the CpG sites in the HIF1α promoter region showed high levels of methylation in the TET1-knockdown JEG3 cells exposed to 3% O_2_ (Fig. 5F), whereas the opposite results were found in the TET1-sgRNA-2-transfected cells, which highly expressed TET1 (Fig. 5G). Moreover, we treated JEG3 cells with the DNA methyltransferase inhibitor 5-aza-Dc (Sigma). Notably, 5-aza-Dc abolished the TET1 knockdown-mediated suppression of HIF1α mRNA expression (Fig. S2). In summary, in cells exposed to 3% O_2,_ TET1 knockdown inhibits the expression of HIF1α and HIF1α target genes, which is accompanied by hyper-methylation of the HIF1α promoter.

### The migration and invasion capacities of JEG3 cells are suppressed by HIF1α knockdown in cells exposed to 3% O_2_

**Figure-6:**
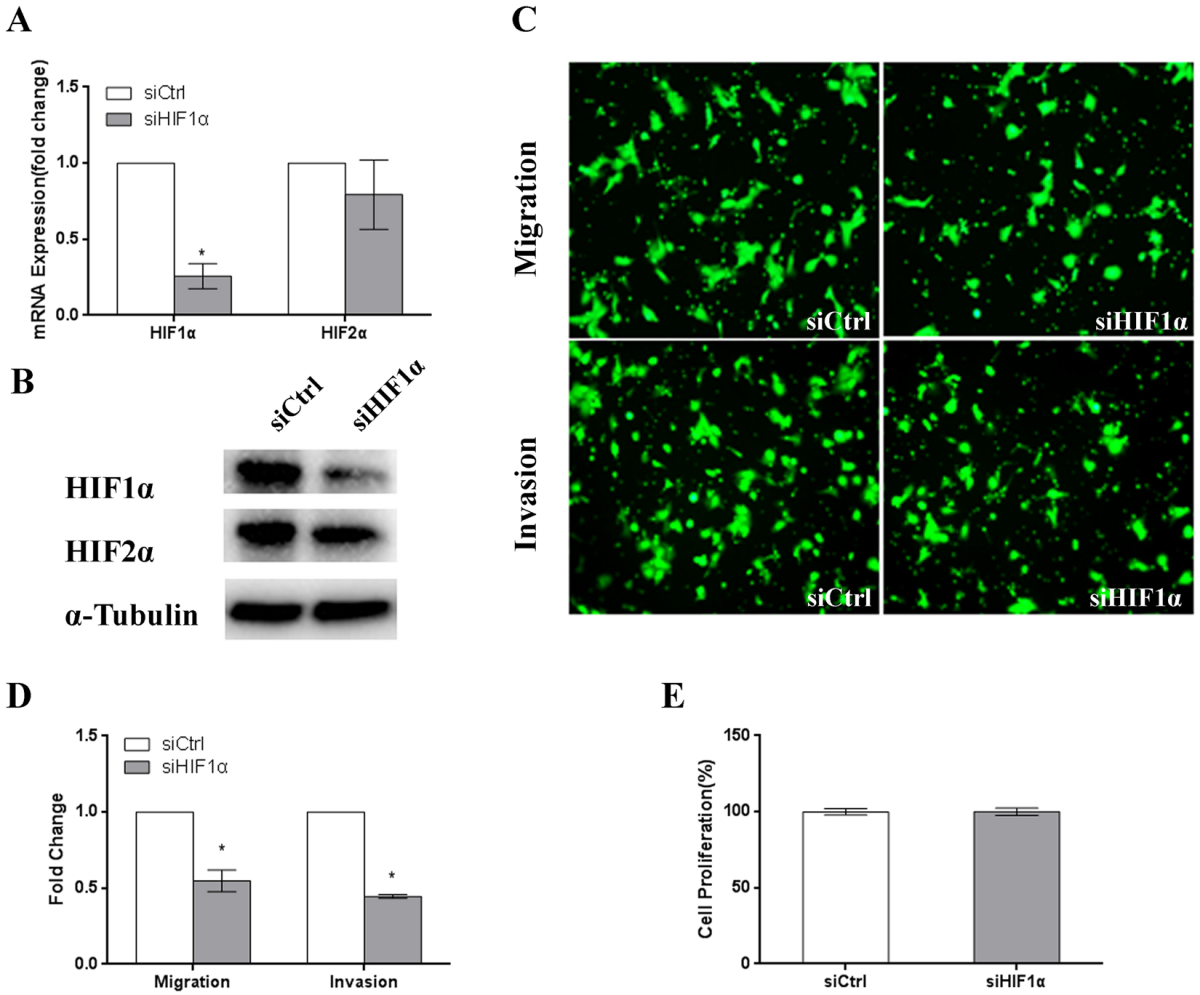
The migration, invasion and proliferation capacities of JEG3 cells are suppressed by HIF1α knockdown in cells exposed to 3% O_2_. (**A**,**B**) Real-time PCR and western blot analyses showing the specific knockdown of HIF1α in JEG3 cells by siRNA. (**C**) The migration and invasion capacities of JEG3 cells transfected with scramble (control) siRNA or HIF1α siRNA. (**D**) The number of migrated or invaded cells was counted and the quantitative data are expressed as the fold change from the control of three independent experiments. (*P < 0.05, **P < 0.01, Student’s t-test) (**E**) The proliferation capacity of JEG3 cells with transfected with scramble (control) siRNA or HIF1α siRNA for 48 h. (not significant, Student’s t-test).

In response to physiological hypoxia, the HIF1α protein is stably expressed in human trophoblasts^27^. During early placentation, HIF1α is localized in trophoblasts and shows high expression at 7–9 week of gestation^12^. In addition, several pro-invasion factors expressed in placentas are HIF target genes that contribute to cell migration and invasion. In our study, TET1 regulated the expression of HIF1α in cells exposed to 3% O_2_. Therefore, to investigate the role of HIF1α in JEG3 cells, a specific siRNA targeting the important hypoxic mediator, HIF1α, was synthesized and transfected into JEG3 cells. First, qPCR (Fig. 6A) and western blot analyses (Fig. 6B) were performed to confirm the efficacy of HIF1α-siRNA in JEG3 cells exposed to 3% O_2_. We found that cell migration and invasion were significantly decreased in the siHIF1α-JEG3 cells exposed to 3% O_2_ (Fig. 6C,D). To identify the effects of HIF1α knockdown on the proliferation of trophoblast cells exposed to 3% O_2_, we detected the proliferation activity of JEG3 cells transfected with HIF1α-siRNA and exposed to 3% O_2_ for 48 h. We found that the proliferation rate was not affected by the deceased expression of HIF1α (Fig. 6E). Because of the complex effects of HIF1α on trophoblast growth including proliferation, apoptosis and the cell cycle^28^, there were various changes in the cell number after siHIF1α transfection^29^. The above data show that HIF1α plays a similar role as TET1 in trophoblast cell migration and invasion.

### Increased HIF1α expression reverses the inhibitory effect of TET1 knockdown on JEG3 cell migration and invasion following exposure to 3% O_2_

**Figure-7:**
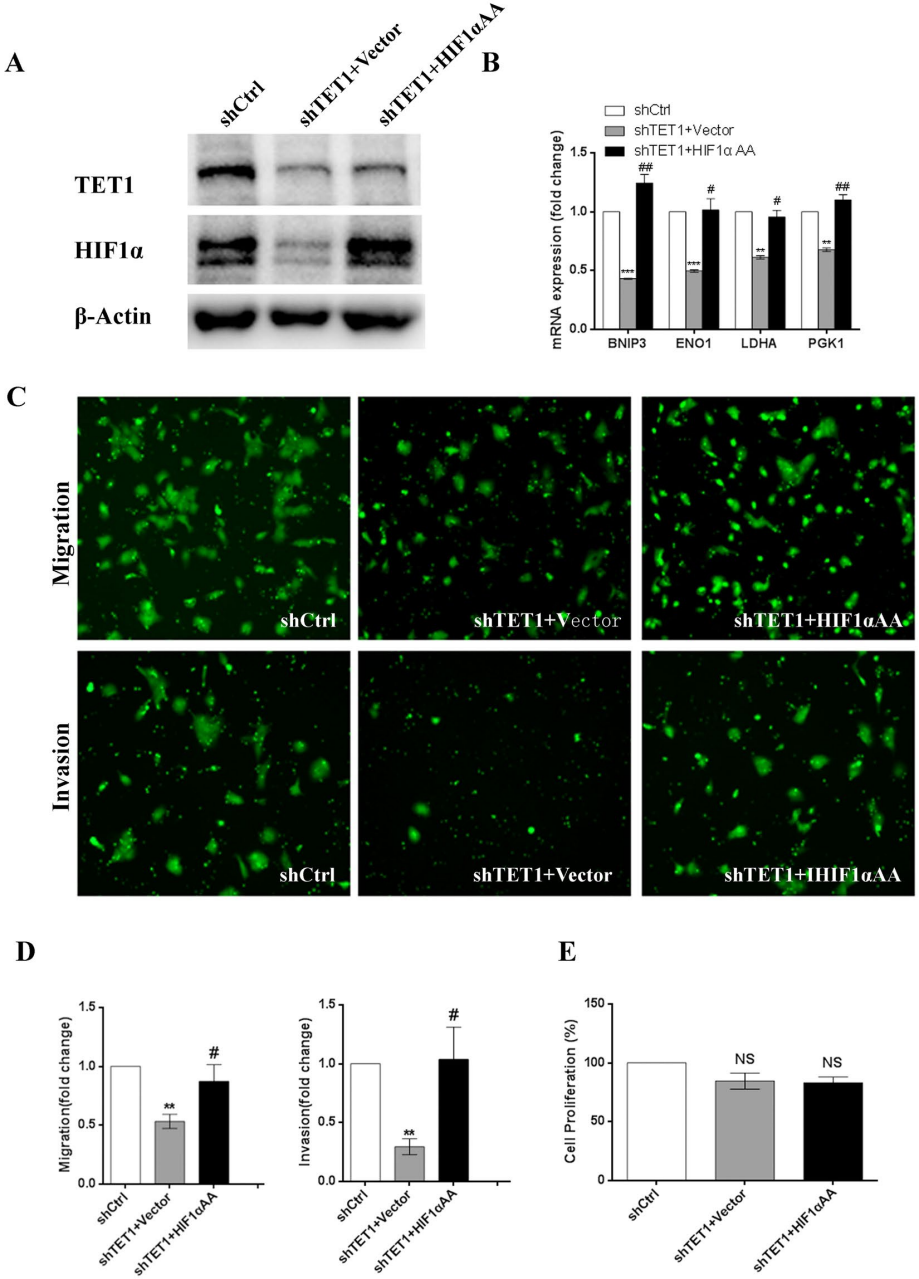
Increased HIF1α expression reverses the inhibitory effect of TET1 knockdown on JEG3 cell migration and invasion following exposure to 3% O_2_. (**A**) The protein expression of HIF1α in scramble control- and shTET1-transfected JEG3 cells transiently transfected with the vector or HIF1αAA for 48 h. (**B**) The mRNA expression of ENO1, LDHA, PGK1 and BNIP3 in scramble control- and shTET1-transfected JEG3 cells transiently transfected with the vector or HIF1αAA for 48 h. (**C**,**D**) The quantitative cell migration and invasion data are expressed as the fold change from the control of three independent experiments. (**P < 0.01, shCtrl vs. shTET1 + Vector; ^#^P < 0.05, shTET1 + Vector vs. shTET1 + HIF1αAA) (**E**) The proliferation capacity in scramble control- and shTET1-transfected JEG3 cells transiently transfected with the vector or HIF1αAA for 48 h.

In well-oxygenated cells, the α subunit of HIF is hydroxylated by enzymes called prolyl-4-hydroxylases (PHDs), which have three isoforms: PHD1, PHD2 and PHD3^30^. Once HIF1α is hydroxylated, it is degraded through a poly-ubiquitination-proteasomal pathway^31, 32^. To obtain constitutively stable HIFα protein expression, ndHIF overexpression plasmids were used, in which the two proline sites of HIF cDNA were changed to alanine (HIF1αAA) according to previously described methods^33^. In cells exposed to 3% O_2_, HIF1αAA was capable of rescuing the HIF1α protein deficiency induced by TET1 knockdown by enhancing HIF1α stability (Fig. 7A). More importantly, HIF1α signaling activity was rescued by inducing the expression of HIF1α target genes (Fig. 7B). Given that TET1 regulated the expression of HIF1α in cells exposed to 3% O_2_, we hypothesized that TET1 plays a role in the regulation of the HIF1α–dependent signaling pathway to effect cell biological functions. Therefore, we performed *in vitro* functional studies using HIF1αAA to increase HIF1α protein expression. As we hypothesized, HIF1αAA was able to overcome the inhibition of cell migration and invasion induced by TET1 knockdown in cells exposed to 3% O_2_ (Fig. 7C,D). However, there was no significant effect on the cell proliferation rates (Fig. 7E), which is consistent with our previous results. These data indicate that increased HIF1α protein expression reverses the inhibitory effect of TET1 knockdown on JEG3 cell migration and invasion following exposure to 3% O_2_.

## Discussion

In early pregnancy, hypoxia is the typical extrinsic factor that regulates trophoblast functions including proliferation, migration and invasion. During this time, TET1 is expressed in trophoblast cells and the transcriptional expression of TET1 is much higher than during the remainder of pregnancy when the blood supply is adequate. In our *in vitro* study, we simulated the physiological hypoxic conditions of early pregnancy and found activation of TET1 in cells exposed to 3% O_2_. We hypothesized that the expression of TET1 in trophoblasts is dependent on physiological hypoxia in early pregnancy. The success of embryo implantation depends on the precise development of the placenta, which involves not only the anchoring of trophoblasts to the uterine wall but also an adequate blood supply for the fetus. In our study, we found that trophoblast cell migration and invasion was up-regulated following exposure to 3% O_2_, which is in agreement with previous reports^7, 8, 10^. However, cell proliferation was significantly decreased under the same O_2_ concentration^8, 10^. These findings indicate that trophoblast migration and invasion are functionally separate from proliferation^34^. All of these results demonstrate that trophoblast invasion and proliferation undergo strict temporal and spatial regulation, which is different from that of tumor cells. The reduced trophoblast invasion of the maternal spiral arterioles during early pregnancy may contribute to pregnancy complications, because this is the most common clinical finding in preeclampia^35^.

*In vitro* studies of cancer cells show that TET1 knockdown mitigates hypoxia-induced migration and invasion^23^ and decreases normal and cancerous breast cell growth and migration^36^. In our study, TET1 was activated following exposure to 3% O_2_, and TET1 knockdown inhibited the migration and invasion capacities of JEG3 cells exposed to 3% O_2_, indicating that TET1 plays a crucial role in the regulation of hypoxia-induced cell migration and invasion in trophoblasts. Recently, TET1 protein expression was shown to be clearly diminished in preeclamptic placentas through the conversion of 5 mC to 5 hmC^37^. Similarly, several studies have shown higher global DNA methylation in the preeclamptic group compared with that in the normal-tension group^38^. Therefore, we hypothesized that TET1 activation by hypoxia is necessary for placentation during early pregnancy and that TET1 depletion may contribute to preeclampsia. HIF1α is the key mediator in the response to hypoxia and contributes to placental differentiation, growth and function. Previous *in vivo* studies showed there is a significant reduction in trophoblast invasion in the placentas of Hif1α^−/−^ mice compared with wild-type placentas^13^. Several pro- and anti-invasion factors expressed in trophoblast cells or the maternal decidua are HIF target genes. Matrix metalloproteinases (MMPs), which are directly required for the degradation of the extracellular matrix, can be activated by HIF1^7, 11, 39^. In *in vitro* studies, HIF1α promotes trophoblast invasion under hypoxia by increasing autophagy^29, 40, 41^, and autophagy participates in pro-invasion mechanisms in studies of glioblastoma cells^42^. A parallel finding in trophoblast cells indicates that trophoblast cell migration/invasion can be inhibited by silencing HIF1α in the JEG3 cell line^43^. In our study, TET1 knockdown was associated with hyper-methylation of the HIF1α promoter, which inhibited the expression of HIF1α in cells exposed to 3% O_2._ This finding is consistent with previous reports in which TET1 was found to be associated with methylation of the gene body, gene promoter and the region around the transcription start site^22, 26^. In our opinion, TET1 regulates cell migration and invasion through the HIF1α signaling pathway. Moreover, increased HIF1α protein expression reverses the inhibitory effect of TET1 knockdown on JEG3 cell migration and invasion following exposure to 3% O_2_.

In conclusion, the activation of TET1 in cells exposed to 3% O_2_ is essential for placentation in normal pregnancy and contributes to the promotion of trophoblast migration and invasion through the HIF1α signaling pathway.

## Materials and Methods

### Sample collection

The first trimester chorionic villi samples (7.43 ± 0.29 weeks, n = 11) were obtained and dissected out immediately after vacuum aspiration from women who underwent a legal termination of an apparently normal early pregnancy. Full-term tissues (39.06 ± 0.11 weeks, n = 12) were obtained from normal placentas after uncomplicated caesarean section deliveries. All samples were stored in liquid nitrogen for RNA extraction and fixed at 4 °C using 4% paraformaldehyde for IHC. All samples were obtained from the Shanghai First Maternity and Infant Hospital. The collection of the tissues was approved by the Shanghai First Maternity and Infant Hospital Scientific and Ethics Committees, and informed consent was obtained from all subjects before sample collection.

### Cell culture and transfection

The trophoblast-like cell line JEG3 was plated and maintained in a culture support center at 37 °C with 5% carbon dioxide/air atmosphere (21% O_2_, standard conditions). After attachment, the cells were maintained in an atmosphere of 21% O_2_ or transferred to a tri-gas incubator with 3% O_2_, 5% CO_2_, and 92% N_2_. To avoid re-oxygenation, cells cultured in 3% O_2_ were harvested immediately using lysis buffer. For TET1 knockdown, lentivirus-containing shRNAs (shTET1-GCAGCTAATGAAGGTCCAGAA, TTCTGGACCTTCATTAGCTGC) were generated in 293 T cells and the JEG3 cells were subsequently infected following standard protocols. TET1 overexpression was achieved by sgRNAs, which combined the target gene promoter region for targeting nuclease-dead Cas9-mediated transcriptional activation of the endogenous mRNA expression of target genes. For TET1 overexpression, lentivirus-containing sgRNAs and dCas9 were co-transfected into JEG3 cells following standard protocols. The lentivirus-containing TET1 sgRNAs (TET1-sgRNA-1, GACAGAGAAGTTGAGAGAGG, CCTCTCTCAACTTCTCTGTC; TET1-sgRNA-2, GGTCGAGAGGGAGTCGAGGA, TCCTCGACTCCCTCTCGACC) and NC-sgRNA were synthesized and obtained from Genechem (Shanghai, China). RNA oligonucleotides specifically targeting HIF1α mRNA were synthesized and obtained from GenePharma (Shanghai, China). HIF1α targeting siRNA (GCCGAGGAAGAACUAUGAATT, UUCAUAGUUCUUCCUCGGCTT) and non-targeting control siRNA were transiently transferred into JEG3 cells using Lipofectamine 2000 (Invitrogen) according to the manufacturer’s protocol. All methods were performed in accordance with the relevant guidelines and regulations. The expression of TET1 or HIF1α in JEG3 cells was further detected by qPCR and western blot analysis.

### Protein extraction, western blotting, RNA extraction, and quantitative real-time PCR

For the extraction of proteins from the cell lines, cell lysis buffer containing protease inhibitors was used. The extracted protein concentration was measured using the BCA Protein Assay Kit (Thermo Scientific). For the western blot analysis, 20 μg of protein extracts from each clone was loaded onto 7.5% SDS-PAGE gels and transferred to polyvinylidene difluoride membranes (Roche Diagnostics). After blocking, the protein was probed with different antibodies (shown in the Supplementary file) and an anti-α-tubulin antibody was used as a loading control. Signals were detected using an ECL chemiluminescence kit (Millipore Corporation, Billerica, MA, USA). Total RNA was isolated using the TRIzol reagent (Life Technologies Corporation, Carlsbad, CA, USA) according to the manufacturer’s recommendations. The 2^−ΔΔCt^ method of relative quantification was used to estimate the copy number of the expression of each gene, and 18 S was selected as the internal control. All of the primers used in the study are listed in the Supplementary file.

### Immunohistochemistry

The paraffin-embedded placental tissues were cut into four-millimeter thick sections. For IHC, the tissue slides were deparaffinized and rehydrated. Before blocking, the slides were pretreated with 3% hydrogen peroxide/methyl alcohol for 10 min to inhibit endogenous peroxidase activity. Non-specific binding sites were blocked with 5% BSA for 30 min and then the tissue sections were probed with the TET1 antibody (1:200, GTX124207, GeneTex) overnight at 4 °C. The sections were then incubated with an HRP-labeled anti-rabbit/mouse IgG polymer (EnVision Detection Kit, Peroxidase/DAB, Rabbit/Mouse, Gene Tech, CA, USA) for 30 min at room temperature. The staining was semi-quantitatively evaluated by assigning an immunoreactivity intensity score (0, null; 1+, low or weak; 2+, moderate; or 3+, high or strong) and the percentage of positively stained cells (0, no cells stained; 0.3, <30% of the cells stained; 0.7, 30–70% of the cells stained; and 1, >70% of the cells stained) was evaluated. The product of these two values was used to calculate the H-score^44^.

### *In vitro* migration/invasion assay

For the *in vitro* migration/invasion assay, we used the 24-Multiwell BD Falcon FluoroBlok Insert System (8.0 μm pores; BD Biosciences, San Jose, CA). The transwell inserts were covered with Matrigel (BD Biosciences) for the invasion assay^10^. In the transwell insert, JEG3 cells were seeded at a density of 5 × 10^4^ for the migration assay or 10 × 10^4^ for the invasion assay in 1% FBS medium (topside of the membrane). The bottom wells of the chamber were filled with 10% FBS medium. After 16 h for the migration assay or 24 h for the invasion assay, the cells that migrated to the bottom of the inserts were stained with calcein AM (0.2 μg/ml; Invitrogen, No. C3100MP) for 30 min and recorded using an inverted microscope mounted with a CCD camera. Four photos were obtained for each well and the results are represented as the fold change in the cell number compared to the number in the control. All experiments were repeated three times.

### Cell proliferation assay

For the cell proliferation assay, JEG3 cells were plated overnight in a 12-well plate at a density of 5 × 10^4^ cells/well for attachment. After the medium was exchanged, the cells were either transferred to 3% O_2_ or maintained in 21% O_2_. The cell proliferation rate is shown as the percentage of cells relative to that in the control group after the cells were digested and counted. Three independent experiments were performed for the cell proliferation assay.

### DNA methylation analysis

Genomic DNA from JEG3 cells was prepared using a genomic DNA extraction kit (Qiagen). Primers for the HIF1α promoter were designed and then bisulfite-PCR amplification was performed to quantitatively assess DNA methylation at the individual CpG sites using the SequenoEpiTYPER system (Sequenom Inc., CA), as described previously. This technique employs base specific cleavage followed by MALDI-TOF mass spectrometry (MS), in which the mass ratio of the cleaved products provides highly quantitative methylation estimates for CpG sites within the target region.

### Statistical analyses

All values in the text are reported as the mean ± SEM, and differences between the means of two groups were evaluated using a two-tailed Student’s t-test. One-way analysis of variance (ANOVA) was used to identify significant differences among multiple groups.

## Electronic supplementary material

Supplementary Information
