# Supplementary material for: Hypoxia-induced TET1 facilitates trophoblast cell migration and invasion through HIF1α signaling pathway
Source: Sci Rep. 2017 Aug 14;7:8077. doi: 10.1038/s41598-017-07560-7 (PMC5556046; doi:10.1038/s41598-017-07560-7)
Supplement: Supplementary file 1 — Supplementary Information [file 41598_2017_7560_MOESM1_ESM.pdf]

**Hypoxia-induced TET1 facilitates trophoblast cell migration and invasion through HIF1α signaling pathway**

**Jingping Zhu[1], Kai Wang[1], Ting Li[2], Jiayu Chen[1], Dandan Xie[1], Xinwen Chang[1], Julei Yao[1], Jinting Wu[1], Qian Zhou[1], Yuanhui Jia[1*]&Tao Duan[2*]**

1 Clinical and Translational Research Center, Shanghai First Maternity and Infant Hospital, Tongji University School of Medicine, Shanghai, 200040, China.

2 Department of Obstetrics, Shanghai First Maternity and Infant Hospital, Tongji University School of Medicine, Shanghai, 201204, China.

*correspondingauthors: Y.J. (email: yuanhui.jia@163.com) &T.D. (email: tduan@yahoo.com)

**Table1: Sequence of the primers for real-time qPCR.**

| **Target** | **Sequence (5' → 3')** |
|---|---|
| 18S | F:GGCGGCGTTATTCCCATGA<br>R:GAGGTTTCCCGTGTTGAG |
| TET1 | F:GAAAGAAGAGGGCTGCGATG<br>R:TGCACGGTCTCAGTGTTACT |
| TET2 | F:ACAGAAGCAAGAACAGCAGC<br>R:AGCTTGCAGGTGGATTCTCT |
| HIF1α | F: GAACGTCGAAAAGAAAAGTCTCG<br>R:CCTTATCAAGATGCGAACTCACA |
| HIF2α | F:CGGAGGTGTTCTATGAGCTGG<br>R:AGCTTGTGTGTTCGCAGGAA |
| PGK1 | F:TGGACGTTAAAGGGAAGCGG<br>R:GCTCATAAGGACTACCGACTTGG |
| LDHA | F:ATGGCAACTCTAAAGGATCAGC<br>R:CCAACCCCAACAACTGTAATCT |
| BNIP3 | F:CCTTCCATCTCTGCTGCTCT<br>R:TCCACTAACGAACCAAGTCAG |
| ENO1 | F:GTGTGGCTCTAACCCTCTGG<br>R:GGACCTTCTGTGGGACCTCT |

**Table2: List of antibodies used in the western blot.**

| Protein | Origin | Dilution | Incubation period |
|---|---|---|---|
| α-Tubulin | GTX628802, Gene Tex. | 1:1,000 | 4 ℃, overnight |
| HIF1α | #3716, CST. | 1:1,000 | 4 ℃, overnight |
| HIF2α | AF2997, R&D. | 1:1,000 | 4 ℃, overnight |
| TET1 | GTX124207, Gene Tex | 1:1,000 | 4 ℃, overnight |
| TET2 | Sc-136926, Santa Cruz. | 1:100 | 4 ℃, overnight |

**Figure-S1: The mRNA expression of HIF1α was not regulated by TET1 by TET1 in cells exposed to 21% $O_2$.** (A) The HIF1α mRNA expression in shCtrl and shTET1 JEG3 cells exposed to 21% $O_2$. (B) The HIF1α mRNA expression in the negative control group and TET1 overexpression group of JEG3 cells exposed to 21% $O_2$.

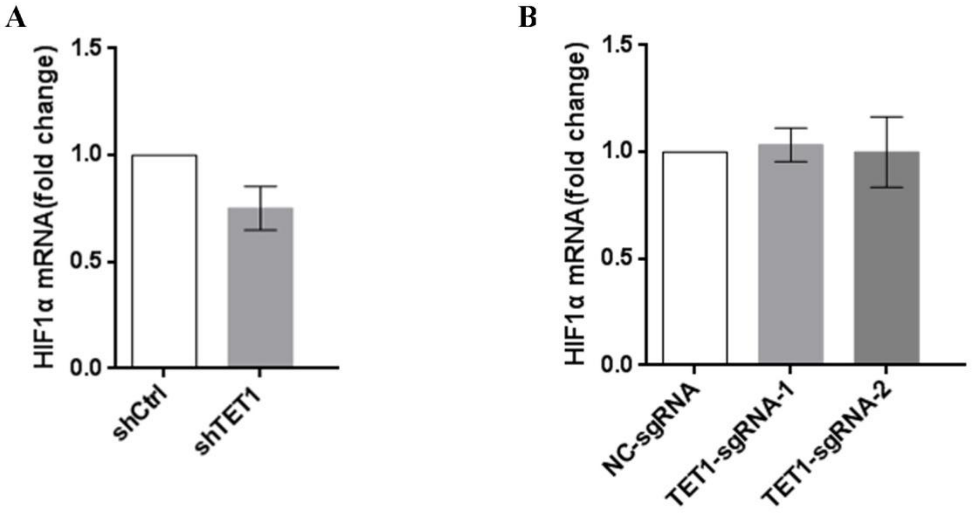


**Figure-S2: The DNA methyltransferases inhibitor (5-aza-Dc, AZA) abolished the TET1-knockdown mediated suppression of HIF1α mRNA levels.** (A) The HIF1α mRNA expression of JEG3 cells treated with different dose of AZA (0, 5, 10 and 20μM) for 72 hours. (*$P<0.05$, one-way ANOVA) (B) The HIF1α mRNA expression in shCtrl and shTET1 JEG3 cells treated with 20μM AZA for 72 hours.

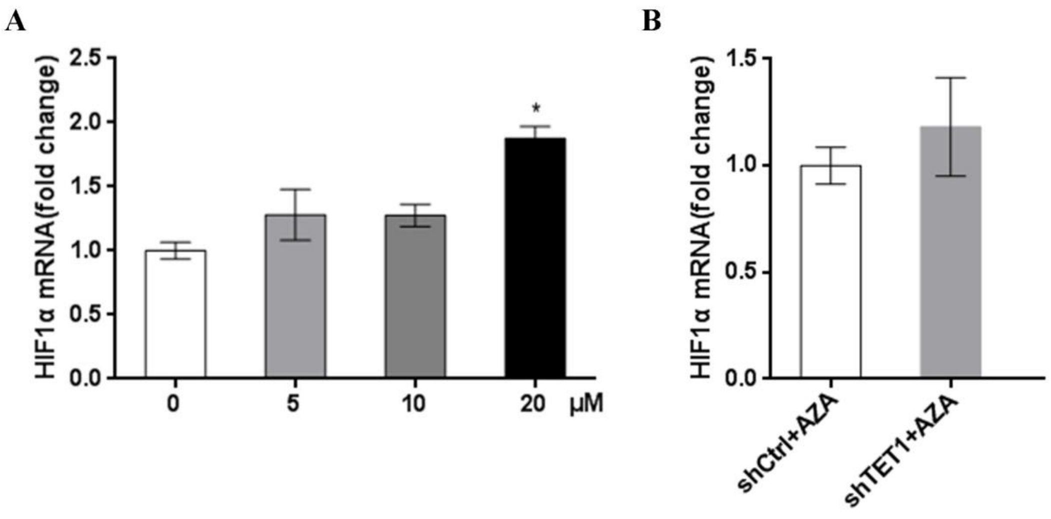
A
B
HIF1α mRNA(fold change)
2.5
2.0
1.5
1.0
0.5
0.0
*
0
5
10
20
μM
HIF1α mRNA(fold change)
1.5
1.0
0.5
0.0
shCtrl+AZA
shTET1+AZA
